# IDO2-AhR axis as central regulator of the kynurenine pathway in glioblastoma

**DOI:** 10.1007/s11060-025-05106-w

**Published:** 2025-06-05

**Authors:** Arnaud Jacquerie, Amanda Macamo, Ann Hoeben, Daniëlle B.P. Eekers, Alida A. Postma, Maxime Vanmechelen, Frederik De Smet, Linda Ackermans, Monique Anten, Maikel Verduin, Kim Severens, Axel zur Hausen, Jan Beckervordersandforth, Martinus P.G. Broen

**Affiliations:** 1 Department of Pathology, GROW School for Oncology and Reproduction, Maastricht University Medical Centre, Maastricht, The Netherlands; 2 Department of Medical Oncology, GROW School for Oncology and Reproduction, Maastricht University Medical Centre, Maastricht, The Netherlands; 3 Department of Radiation Oncology (Maastro), GROW School for Oncology and Reproduction, Maastricht University Medical Centre, Maastricht, The Netherlands; 4 Department of Radiology and Nuclear Medicine, Mental Health and Neuroscience research institute, Maastricht University Medical Centre, Maastricht, The Netherlands; 5 Laboratory for Precision Cancer Medicine, Translational Cell and Tissue Research Unit, Department of Imaging and Pathology, KU Leuven, Leuven, Belgium; 6 LISCO—KU Leuven Institute for Single Cell Omics, KU Leuven, Leuven, Belgium; 7 Department of Medical Oncology, University Hospitals Leuven, Leuven, Belgium; 8 Department of Neurosurgery, School for Mental Health and Neuroscience, Maastricht University Medical Centre, Maastricht, The Netherlands; 9 Department of Neurology, GROW School for Oncology and Reproduction, Maastricht University Medical Centre, Maastricht, The Netherlands

**Keywords:** Kynurenine, IDO, TDO2, AhR, Glioblastoma

## Abstract

**Purpose:**

Upregulation of the Kynurenine Pathway (KP) in Glioblastoma (GBM) plays an important role in driving its treatment-resistant immunosuppressive microenvironment. Factors driving this exaggerated pathway remain poorly understood. Our aim was to explore the correlation between key KP markers; IDO1, IDO2, TDO2, its primary effector target aryl hydrocarbon receptor (AhR) and a comprehensive set of clinical- and tumour characteristics.

**Methods:**

Tissue samples from 108 newly diagnosed GBM patients were analyzed for the expression of TDO2, IDO1, IDO2, and AhR using immunohistochemistry and QuPath software. Exploratory analyses were conducted to evaluate correlations between KP marker expression and clinical, radiological, and molecular data.

**Results:**

IDO1 expression was primarily correlated with inflammatory blood markers, while TDO2 was correlated with patient age, gender, smoking habit and medication use. In contrast, AhR and IDO2 demonstrated hardly any correlations with clinical or tumour characteristics. Notably, IDO2 exhibited a strong association with AhR expression and tumour cell density, with no observed correlation between AhR and either IDO1 or TDO2.

**Conclusions:**

We validated the inflammatory influences on IDO1 expression and found that TDO2 was mostly correlated with medication and patient characteristics. We could not confirm IDO1 and TDO2 as most prominent drivers of AhR activity in the KP. However, we found a strong correlation between IDO2-AhR which may be responsible for the sustained and enhanced immunosuppression within the tumour microenvironment. This could explain recent failures of IDO1 and TDO2 antagonists and might redirect future studies to intervene in the kynurenine-AhR-IDO2 axis.

**Supplementary Information:**

The online version contains supplementary material available at 10.1007/s11060-025-05106-w.

## Introduction

Glioblastoma (GBM) is the most aggressive and lethal form of primary brain cancer, with 5-year survival rates of less than 5% when treated [1]. Their treatment resistance is mostly ascribed to inter-and intratumoural heterogeneity combined with an immunosuppressive microenvironment [2]. In the last decade, kynurenine pathway (KP) has gained considerable attention as a central regulator of the immune microenvironment in GBM [3–5]. The KP is a metabolic route that generates various bioactive compounds through breakdown of the essential amino acid tryptophan (TRP) [6, 7]. Briefly, TRP is converted into kynurenine (KYN), the central metabolite of the KP, by a trio of enzymes: tryptophan 2,3-dioxygenase (TDO2), indoleamine 2,3-dioxygenase 1 (IDO1) and indoleamine 2,3-dioxygenase 2 (IDO2) [7]. KYN can be further processed into several neuroactive metabolites [5, 7]. The KP is intensely dysregulated in GBM [5, 7, 8]. There is substantial local depletion of TRP, and KP metabolizing enzymes are overexpressed in GBM, ultimately leading to the accumulation of KYN metabolites [7, 9]. KP is an endogenous ligand of the aryl hydrocarbon receptor (AhR), a cytoplasmic transcription factor linked to carcinogenesis [10]. The AhR serves as a master regulator in the differentiation and effector functions of lymphocytes and macrophages [8, 10–14]. Consequently, activation of AhR by KYN is thought to play a major role in creating an immunosuppressive environment in GBM [8, 10–14].

We recently discovered that protein expression of IDO1, IDO2, TDO2 and AhR are independent prognostic factors in GBM [15]. However, the contributing factors driving the upregulation of the KP in GBM are currently unknown. Existent literature centers predominantly around IDO1 in GBM. Despite promising efficacy in preclinical models, IDO1 inhibitors failed to live up to their expectations in clinical trials. The precise reasons behind the inefficacy of IDO1 inhibitors remain unclear [16–18]. Our aim is to investigate the KP with a broadened scope, exploring the IDO1, IDO2, TDO2 and AhR interplay with both clinical as well as tumour characteristics. By doing so, we hypothesize that we create a more thorough understanding of factors influencing the KP in GBM patients. This will help in our understanding of the complex role of KP in GBM and could ultimately aid in developing and enhancing treatment strategies targeting KP.

## Methods

### Patient selection

**Fig. 1:**
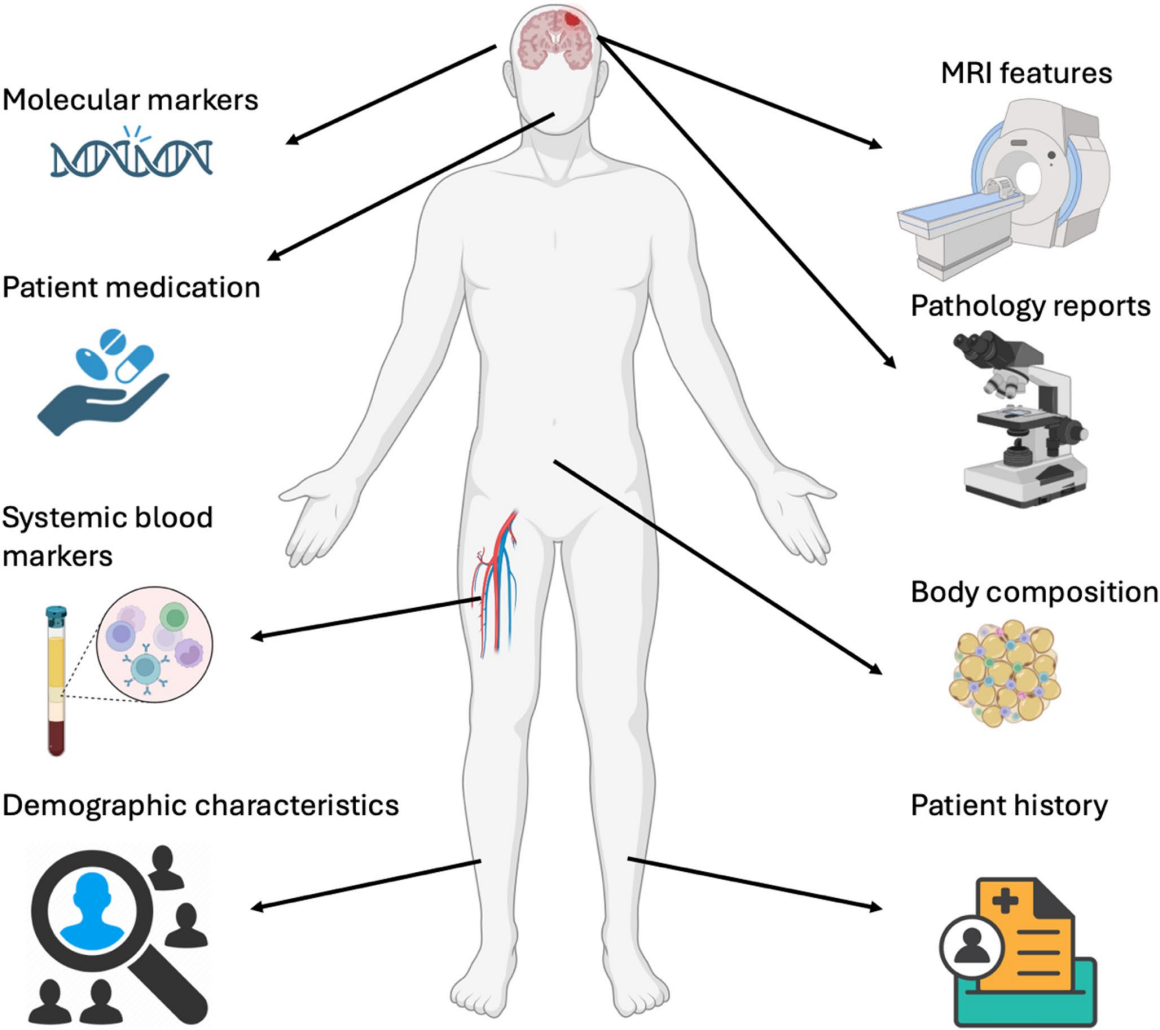
Variables analyzed in relation to IDO1, IDO2, TDO2 and AhR expression in glioblastoma

Patient samples with accompanying clinical, tissue, and genotypic data from 135 GBM patients diagnosed or treated at Maastricht University Medical Center+ (MUMC+, Netherlands) between 2006 and 2014 were available in a pre-existing glioma database [19]. Patients with newly diagnosed GBM (WHO grade 4), isocitrate dehydrogenase gene 1 or 2 (IDH1/2) wildtype, regardless of MGMT methylation status, were included. IDH-mutant GBMs and recurrent cases were excluded to reduce biological heterogeneity and maintain a consistent cohort of newly diagnosed, IDH-wildtype GBM, which reflects the current WHO diagnostic criteria for GBM. These exclusions were necessary to avoid confounding due to known differences in TRP metabolism and immune contexture associated with IDH mutations and prior therapy. To investigate the interplay between the expression of IDO1, IDO2, TDO2, and AhR with various clinical and tumour characteristics, we conducted exploratory analyses utilizing the comprehensive clinical data available (Fig. 1, for an overview see Supplement 1).

The study was reviewed by the Medical Ethical Committee. Patient consent was waived for our retrospective study by the accredited Medical Review Ethics Committee of Maastricht UMC+(METC record 2022–3327).

Patient material was used according to the Code for Proper Secondary Use of Human Tissue (Federation of Medical Scientific Societies, The Netherlands; 2013). All experiments were performed in accordance with relevant guidelines and regulations.

### Demographic characteristics and body composition

Patient characteristics and relevant clinical data were extracted from medical records, including age at diagnosis, gender, body mass index (BMI), and Eastern Cooperative Oncology Group (ECOG) performance status scale. Additionally, body composition metrics, including visceral adipose tissue (VAT) and skeletal muscle index (SMI), were assessed at the L3 vertebral level using abdominal CT scans and analyzed with Slice-O-Matic software v5.0 [20]. These metrics were incorporated into our exploratory analyses, as outlined in a previous study.

### Patient history and medication usage

Patient medical history was extracted from medical records, with a focus on key factors such as previous malignancies, diabetes, hypertension, and smoking/alcohol history. Additionally, data on commonly used medications in GBM patients, such as corticosteroids and anticonvulsant therapy, were collected. For a complete overview, see Supplement 1.

### Systemic blood markers

Data on routinely measured preoperative hematological markers were retrieved from the patients’ medical records. These included but were not limited to, leukocyte and platelet counts, providing insights into the patients’ systemic inflammatory and coagulation status prior to GBM surgery. Additional available blood markers, such as hemoglobin levels, lymphocyte counts, and C-reactive protein (CRP) levels, were also collected. A comprehensive overview of all systemic blood markers analyzed is shown in Supplement 1.

### Tumour characteristics

DNA was isolated from formalin-fixed, paraffin-embedded GBM tissue for next-generation sequencing (NGS, Ion Torrent Cancer Hotspot panel v2Plus) and MGMT methylation status was assessed using methylation-specific multiplex ligation-dependent probe amplification (MS-MLPA) as previously described [21]. Data on the following molecular markers was generated: IDH1/2 mutation (yes/no), TERT promotor mutation (yes/no), MGMT-methylation (yes/no), EGFR mutation or amplification (yes/no), PTEN mutation (yes/no), TP53 mutation (yes/no), PIK3CA mutation (yes/no) and MDM2 amplification (yes/no). Additionally, MRI characteristics were assessed and scored using the Visually Accessible Rembrandt Images (VASARI) feature set [22] (see Supplement 1 for details).

### Immunohistochemical staining and digital pathology-based scoring of kynurenine pathway markers

**Fig. 2:**
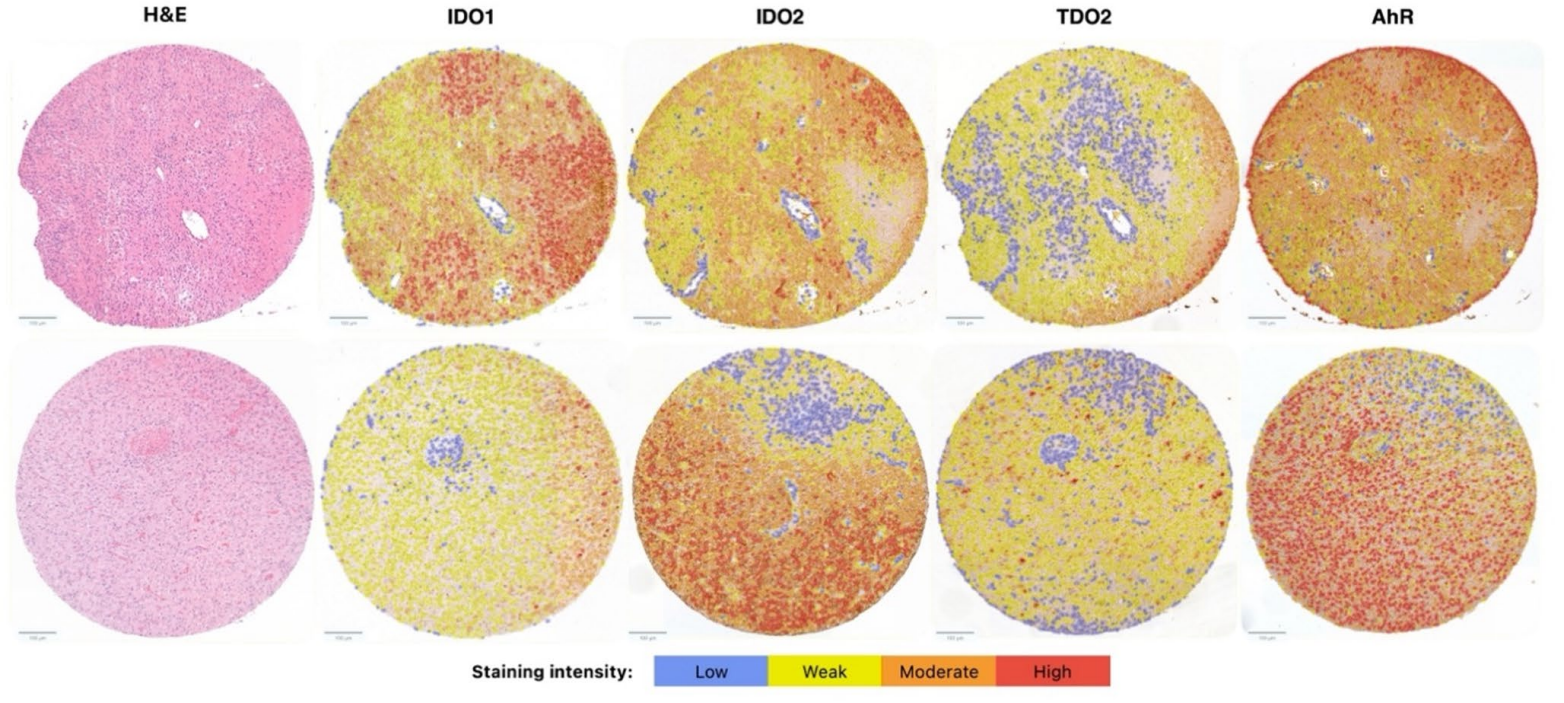
Images of two representative glioblastoma TMA cores demonstrate hematoxylin and eosin (H&E) staining alongside immunohistochemical (IHC) detection of IDO1, IDO2, TDO2, and AhR, with immunoscoring performed using QuPath. Cells are color-coded based on the expression levels of Kynurenine pathway markers, determined by mean DAB staining intensity: cytoplasmic staining for IDO1, IDO2, and TDO2, and nuclear staining for AhR. Expression levels are represented as follows: blue (negative), yellow (weak positive), orange (moderate positive), and red (strong positive). Abbreviations: H&E (hematoxylin and eosin), TDO2 (tryptophan 2,3-dioxygenase), IDO1 (indoleamine 2,3-dioxygenase 1), IDO2 (indoleamine 2,3-dioxygenase 2), and AhR (aryl hydrocarbon receptor)

GBM tumour samples were processed into paraffin-embedded tissue-micro arrays (TMA). Three cores of 0.6 mm diameter per patient were selected and 3 μm-thick TMA sections were cut. Immunohistochemical staining and digital pathology-based scoring of KP markers were conducted following an established protocol described previously [23]. Briefly, antigen retrieval was performed to enhance epitope exposure. Subsequently, sections were incubated with primary antibodies targeting IDO1, IDO2, TDO2 and AhR, followed by a signal amplification step using the Dako FLEX + rabbit or mouse Linker antibody and visualized through chromogenic reaction. Counterstaining with hematoxylin was applied to facilitate nuclear visualization. Stained TMA slides were scanned, and QuPath version v0.2.2, a validated software for digital pathology image analysis was used for semi-automatic digital quantification of IDO1, IDO2, TDO2 and AhR expression (Fig. 2) [24]. The histochemistry score (H-score) was computed to quantify the expression of the KP markers [24, 25].

### Explorative tumour immune microenvironment analysis

To investigate potential spatial relationships between immune infiltration and KP marker expression in GBM, we conducted an exploratory multiplex immunofluorescence analysis on TMA cores. Immune and tumour cell populations were identified and segmented at single-cell resolution using MILAN cyclic immunofluorescence staining and a validated computational pipeline, as previously described [26]. Quantification of positive cells was performed using QuPath. The density of each immune cell subtype, including macrophages (CD68 + TMEM119−), microglia (CD68 + TMEM119+), T cells (CD3+), dendritic cells (CD1c+), and tumour cells was calculated as a percentage of all nucleated cells per TMA core. Subsequently, these immune metrics were correlated with the expression levels of KP markers (IDO1, IDO2, TDO2, AhR) as well as with tumour cell density.Due to technical constraints, these analyses were performed on tissue sections located a few micrometers deeper than the sections used for KP marker quantification. As such, they represent adjacent but not identical tumour regions. In addition, while this approach allowes estimation of major immune populations, it does not enable detailed cell-type or functional profiling.

### Statistical analyses

Statistical analysis was performed using SPSS version 29 (IBM Corporation, Armonk, NY, USA). Descriptive statistics were computed for key variables within a cohort of 108 patients. Moreover, normality tests, such as the Shapiro-Wilk test, were conducted to assess the distributional characteristics of the data.

X-tile was used to determine optimal cut-off values for H-scores of IDO1, IDO2, TDO2 and AhR and divide patients into a ‘low’ or a ‘high’ expression group per KP marker [27]. For dichotomous variables, point-biserial correlation analyses were conducted, followed by a chi-square test to compare the high versus low expression groups of the KP markers. In the case of small counts in 2 × 2 tables, the Yate’s Continuity Correction was applied. Binary logistic regression analysis was performed to determine the predictive value of the explored variables on KP marker expression. For continuous variables, either Pearson or Spearman correlation analyses were employed, depending on normality. Mean differences were assessed using the Mann-Whitney U-test or the student t-test, depending on the distribution of the data. In the case of categorical variables with multiple categories, the Kruskal-Wallis or ANOVA test was performed, followed by pairwise comparisons. A *P*-value < 0.05 was considered statistically significant.

## Results

### Patients

**Fig. 3:**
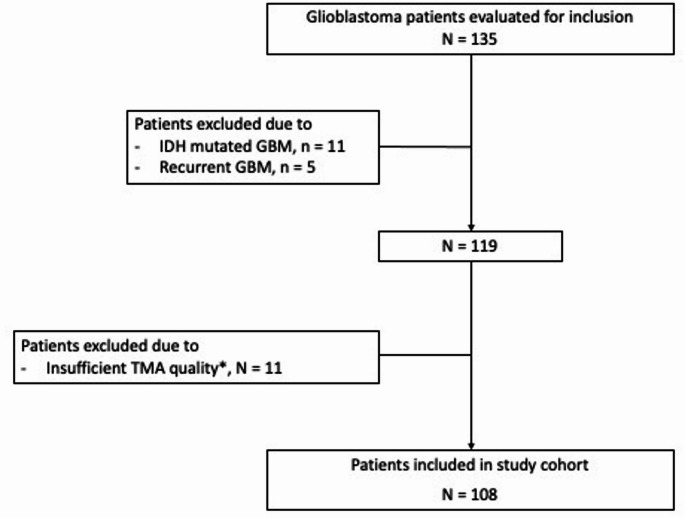
Flowchart of the patient selection process

Out of the 135 GBM patients assessed for inclusion, 16 were excluded for the following reasons: 11 due to IDH mutation and 5 due to recurrent GBM. Additionally, the TMA quality was deemed inadequate for eleven patients, leading to their exclusion from further analysis. The reasons for TMA exclusion included misfolded core tissue (*n* = 2), unrepresentative areas for tumour tissue due to the presence of large blood vessels or necrosis (*n* = 5), and insufficient tissue (*n* = 2). The final study cohort comprised 108 patients (Fig. 3), general cohort characteristics are shown in Table 1.

**Table 1:** Cohort characteristics

Variables	Study Cohort (*n* = 108)	
**Age at diagnosis**,** years**,** mean (SD)**	60.3 (12.5)	
**Gender**,** n (%)**		
Male	64 (59.3)	
Female	44 (40.7)	
**BMI**,** mean (SD)**	26.2 (4.0)	
**ECOG score at baseline**,** n (%)**		
0 or 1	101 (93.5)	
≥ 2	4 (3.7)	
**Overall survival**,** months**,** mean (SD)**	14.5 (10.8)	
**Stupp protocol completed**,** n (%)** Yes No Missing	45 (41.7) 62 (57.4) 1 (0.9)	
**MGMT hypermethylation**,** n (%)**		
Yes	31 (28.7)	
No	76 (70.4)	
Not available due to technical reasons	1 (0.9)	

*ECOG* Eastern Cooperative Oncology Group Performance Status, *MGMT 06-*methylguanine-DNA-methyltransferase, *n* number of patients

### Demographic characteristics and body composition

The correlation between main demographic characteristics and the expression of KP markers is presented in supplement 2. Significant correlations are summarized in Table 2. Age at diagnosis was found to be associated with TDO2 expression (ρ = 0.288, *P* < .05). There was a significant difference in mean age at diagnosis between patients with high (62.2 ± 10.9) and low TDO2 expression ([56.1 ± 11.8], U = 1079, *P* < .05). Gender was also significantly associated with TDO2 expression (χ² (1) = 6.286, *P* < .05). The odds of having high TDO2 expression were one-third lower for males compared to females (OR = 0.300, 95% C.I.: 0.115-0.783, *P* < .05).

The association between body composition and KP markers expression was explored (supplement 2). Only visceral adipose tissue (VAT) was significantly associated with high IDO1 expression. The odds of having elevated IDO1 expression were 1.015 higher (OR 1.015, 95%C.I.: 1.003–1.026, *P* < .05) for each cm^2^ increase in visceral adipose tissue.

### Patient history and medication usage

Exploratory analyses were conducted to investigate the relationship between patient history and KP marker expression (for a complete overview of variables, see Supplement 3). Smoking history was the only factor found to be significantly associated with increased TDO2 expression (χ²(1) = 8.111, *P* < .01).

The correlation between medication usage and KP marker expression is detailed in supplement 4, with significant findings summarized in Table 2. Dexamethasone use was associated with elevated TDO2 expression in GBM patients (χ²(1) = 5.571, *P* < .05), though no dose-response relationship was observed across the cohort. Statin use was similarly linked to higher TDO2 expression (χ²(1) = 6.338, *P* < .05).

Additionally, anticonvulsant therapy was correlated with increased AhR expression (χ²(1) = 7.307, *P* < .01). The type of anticonvulsant used did not significantly influence these findings, suggesting consistency across different anticonvulsant medications.

Due to the small number of patients using antipsychotics (*n* = 4), thyroid hormone replacements (*n* = 3), and metformin (*n* = 3), no correlation analyses were performed for these medications.

### Systemic blood markers

The Spearman rank correlation analyses between systemic blood markers and KP markers are presented in supplement 5, with significant correlations in Table 1. High preoperative blood leukocyte count was significantly associated with stronger IDO1 expression (*r* = .319, *P* < .05). The same correlation was observed between IDO1 H-score and preoperative CRP (*r* = .301, *P* < .05). Remarkably, CRP was negatively associated with AhR expression (*r* = −.349, *P* < .05).

Our explorative analyses also revealed moderate correlation between preoperative serum creatinine and IDO1 expression (*r* = .301, *P* < .05). Furthermore, there was a negative correlation between preoperative platelets count and IDO1 expression (*r* = −.290, *P* < .05).

### Tumour characteristics

Correlation analysis results between molecular markers obtained from NGS and MS with the expression of KP markers are available in supplement 6. No associations were found between the expression of TDO2, IDO1, IDO2 and AhR across TERT, MGMT, PTEN, TP53, EGFR mutation or amplification status. Due to the limited number of patients with MDM2 amplification (*n* = 6), and PIK3CA mutation (*n* = 7), no statistical analyses were conducted.

The correlation between MR imaging characteristics of GBM using the VASARI feature set and the expression of KP markers is shown in Supplement 7. The sole factor linked to KP markers, namely elevated TDO2 expression, was the tumour location (left, right, bilateral) (T(2) = 6.024, *P* < .05). TDO2 expression was significantly higher in tumours located in either the left or right hemisphere compared to those with bilateral involvement.

**Table 2:** Influence of clinical characteristics on the expression of kynurenine pathway markers. Summary of significant correlation analyses

Variable	High Group (*N*)	Low Group (*N*)	Spearman Correlation, *r* (*P*)	Chi-square, Cramér’s V (*P*)	Sign. level
*TDO2 expression*					
**Age at diagnosis**	NA	NA	0.288 (0.009)	NA	**
**Gender**					
Male	21	30	NA	0.279 (0.012)	*
Female	21	9			
**Smoking**					
Yes	22	13	NA	0.348 (0.004)	**
No	9	23			
**Use of dexamethasone**					
Yes	30	20	NA	0.267 (0.018)	*
No	9	19			
**Use of statins**					
Yes	12	3	NA	0.284 (0.011)	*
No	28	36			
*IDO1 expression*					
**Leukocyte count**	NA	NA	0.319 (0.010)	NA	*
**CRP**	NA	NA	0.301 (0.045)	NA	*
**Creatinine**	NA	NA	0.301 (0.017)	NA	*
**Platelets**	NA	NA	− 0.290 (0.027)	NA	*
*IDO2 expression*					
**NA**					
*AhR expression*					
**Anticonvulsants**					
Yes	6	10	NA	0.297 (0.007)	**
No	34	29			
**CRP**	NA	NA	− 0.017 (0.876)	− 0.349 (0.014)	*

Kynurenine pathway markers were analyzed in relation to clinical characteristics. Associations with categorical variables were tested using chi-square and Cramer’s V, while Spearman correlation was used for continuous variables. Significance was set at *P* < 0.05. Patients were classified as ‘high’ or ‘low’ based on TDO2, IDO1/2, and AhR H-scores relative to established cut-offs. TDO2: tryptophan 2,3-dioxygenase; IDO1/2: indoleamine 2,3-dioxygenase 1/2; AhR: aryl hydrocarbon receptor; *P < .05, **P < .01

### IDO1, IDO2, TDO2 and AhR

The correlation among TDO2, IDO1, IDO2 and AhR expression can be found in Supplement 8. IDO2 expression was found to have a robust correlation with AhR expression (*r* = .532, *P* < .001, Supplement 9). No significant correlations were observed between TDO2, IDO1, and AhR expression.

Next, we explored the percentage of tumour cells per TMA core using QuPath. Tumour cells were identified and defined in QuPath using a Random Trees classifier, trained by an experienced neuropathologist (JB) who annotated representative tumour regions across a subset of TMA cores. Interactive feedback on classification performance was provided through markup images. The trained classifier then quantified absolute tumour cell counts and percentages across all cores. We averaged the results across three tumour cores per patient. This percentage was then correlated with the H-scores of various KP markers (Supplement 9). IDO2 expression demonstrated a strong correlation with the percentage of tumour cells (*r* = .448, *P* < .001, Supplement 10). In contrast, no correlations were found between the percentage of tumour cells and the expression of TDO2, IDO1, and AhR.

### IDO2 and AhR expression correlate with immune cell exclusion in tumour-dense regions

To assess spatial relationships between immune cell infiltration and KP marker expression, we performed an exploratory multiplex immunofluorescence analysis on GBM tumour cores.

We observed that IDO2 expression was significantly positively correlated with the percentage of tumour cells (*r* = .455, *p* < .001) and inversely correlated with macrophage infiltration (*r* = –.260, *p* = .001) (Supplement 10). Tumour cell density itself was negatively associated with both macrophages (*r* = –.558, *p* < .001) and T cells (*r* = –.339, *p* < .001), suggesting that immune exclusion is more pronounced in densely cellular regions. In contrast, AhR expression showed no significant correlations with any of the quantified immune cell types, possibly reflecting its widespread expression across multiple cell populations.

## Discussion

Our exploratory study shows that IDO1 tumour expression is mainly correlated with inflammatory (blood) markers and TDO2 expression predominantly with patient characteristics such as age, gender, smoking habit and medication use in newly diagnosed GBM patients. In contrast, both AhR and IDO2 had hardly any correlation with patient, blood or tumour characteristics. Nonetheless, IDO2 expression was closely aligned with AhR expression and tumour cellularity, while no such association was found between AhR and either IDO1 or TDO2.

The immunosuppressive role of IDO1 within the Kyn-AhR axis in GBM tumour environment has been extensively studied in the past decade [18, 28–30]. Although normally expressed at low levels in the CNS, IDO1 expression increases with glioma grade and is further upregulated by inflammatory stimuli [7, 18, 31]. In line with this, our data linked IDO1 expression to inflammatory indicators such as leukocyte count, CRP, and VAT [18, 32]. VAT is considered a key source of pro-inflammatory molecules, contributing to chronic inflammation [33, 34].

TDO2, primarily expressed in the liver, plays a central role in systemic TRP metabolism [8]. In GBM, it is also a major TRP-degrading enzyme [10]. Consistent with its systemic regulatory role, we found that TDO2 expression correlated with age, smoking, and statin or corticosteroid use. Notably, corticosteroids were associated with increased TDO2 expression, contrasting earlier in vitro findings by Platten et al. [8], but consistent with studies showing glucocorticoid-induced TDO2 expression in liver and hippocampal tissue [35, 36]. These contrasting findings underscore the need for further investigation into the interaction between TDO2 and glucocorticoids in vivo.

Research on IDO2 in GBM is limited, but it has been implicated in immune evasion through the Kyn–AhR pathway in multiple tumour types [37]. Several studies suggest that AhR selectively induces IDO2 expression [37–40], and recent findings from COVID-19 models highlight a potential IDO2-driven positive feedback loop sustaining AhR activation in severe disease [41, 42]. In our study, IDO2 showed no significant associations with clinical, inflammatory, or tumour characteristics, possibly due to its more restricted, basal expression profile compared to IDO1. Importantly, IDO2 expression closely mirrored that of AhR and tumour cell density, reinforcing a potential regulatory link between the two.

Interestingly, no correlation was observed between IDO1 or TDO2 and AhR expression, despite AhR’s established roles in tumour progression and immune modulation [8]. AhR is broadly expressed and can be activated by a variety of ligands, including KYN, kynurenic acid, and microbial metabolites [7, 43]. The absence of association with IDO1 or TDO2 may indicate alternative activators within the tumour microenvironment. For instance, recent evidence from Sadik et al. suggests that IL4I1 is a dominant AhR-activating enzyme in GBM, more frequently associated with AhR activity than IDO1 or TDO2 [44]. Although IL4I1 was not assessed in our study, the co-expression of IDO2 and AhR might suggests that IDO2 could play a more stable role in sustaining AhR activation in this setting.

**Fig. 4:**
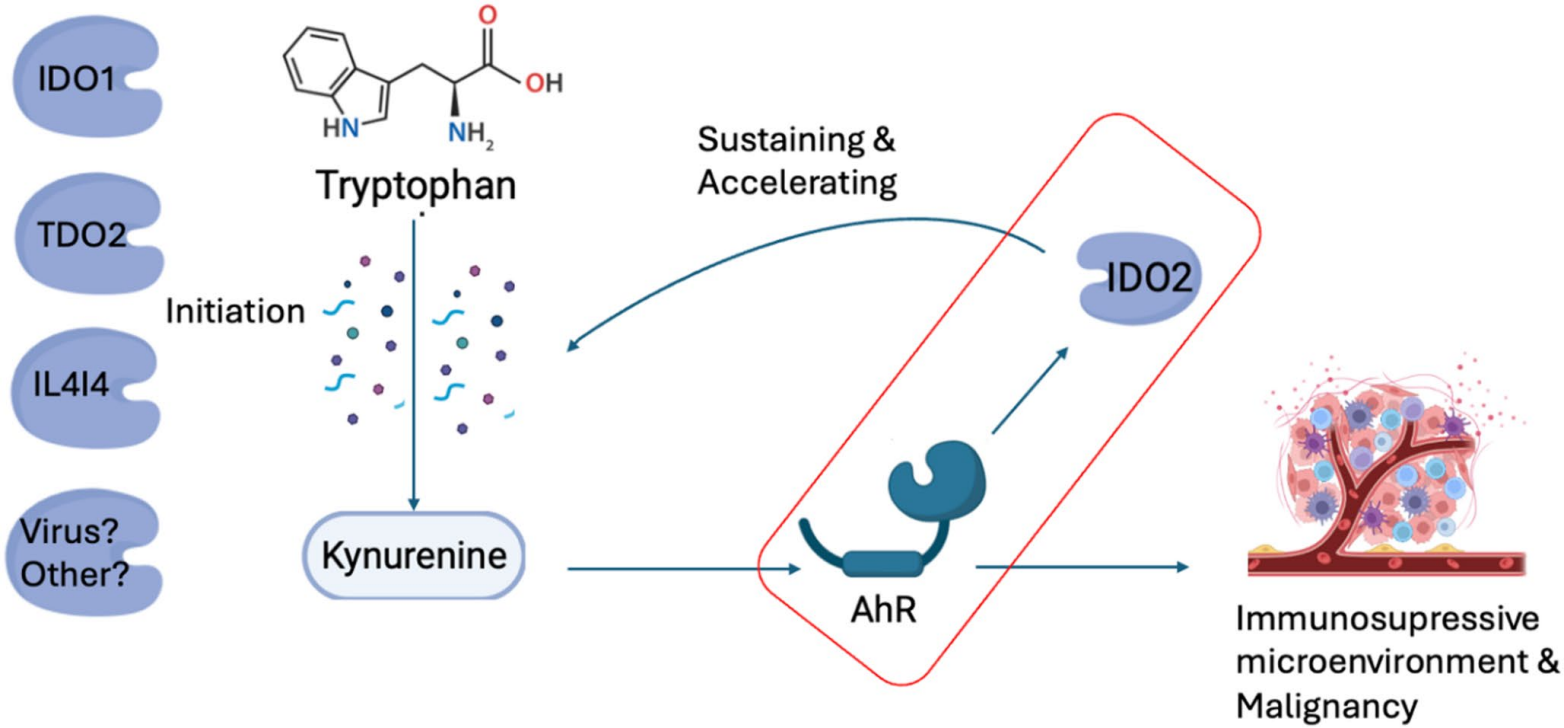
The IDO2-AhR Axis as Central Driver of the Kynurenine Pathway in Glioblastoma

To better understand these spatial dynamics of IDO2–AhR signaling, we conducted an explorative multiplex immunofluorescence on selected tumour and immune cell markers. Despite limitations, including analysis of adjacent (non-identical) tumour sections and a restricted immune panel, we found that IDO2 expression was enriched in tumour-dense regions with reduced macrophage and T cell presence. AhR displayed a similar spatial distribution of IDO2, supporting a potential role for the IDO2–AhR axis in localized immune exclusion. These preliminary findings are consistent with the hypothesis that the IDO2–AhR axis may contribute to localized immune exclusion in the GBM microenvironment, but should be interpreted with some caution. Notably, as our cohort included newly diagnosed, treatment-naïve GBM cases, the tumours likely reflect a later stage of disease progression characterized by established immunosuppressive pathways. The observed co-expression of IDO2 and AhR, and the absence of associations between AhR and IDO1 or TDO2, may indicate a shift toward IDO2–AhR–driven immune suppression in advanced tumours (Fig. 4). Earlier tumour stages may involve different KYN-pathway dynamics, including IDO1- or TDO2-driven KYN production.

At the molecular level, transcriptomic studies have reported low or absent IDO2 RNA levels in GBM, which contradicts with our findings [45]. One possible explanation is that differences between mRNA and protein data are common and may reflect post-transcriptional regulation, protein stability, or tumour microenvironmental effects. Bulk RNA sequencing may also miss low-abundance transcripts restricted to specific tumour regions or subpopulations [46].

Other limitations should be noted. This retrospective study relied on electronic medical records, which may include inaccuracies or missing data, especially regarding medication history. Peri-tumoural tissue was not assessed, and spatial analysis was limited to selected markers on non-identical sections. The single-timepoint design precludes assessment of temporal changes in KP expression, treatment response, or progression. IDH-mutant and recurrent GBMs were excluded to reduce heterogeneity, limiting generalizability. Although our data suggest a regulatory link between AhR and IDO2, mechanistic validation through functional studies is needed. Lastly, no correction for multiple testing was applied due to the exploratory nature of our analyses, underscoring the need for validation in larger, independent cohorts.

In conclusion, we confirmed that IDO1 expression is associated with inflammation and that TDO2 correlates with systemic and clinical characteristics. Neither enzyme, however, showed strong associations with AhR. Instead, we observed consistent co-expression between IDO2 and AhR, particularly in tumour-dense regions, suggesting a role in sustaining immunosuppression. This may contribute to the limited success of current KP-targeting trials and highlights the need for further studies to unravel the KYN–AhR–IDO2 axis and develop more effective therapeutic strategies.

## Electronic supplementary material

Below is the link to the electronic supplementary material.

Supplementary Material 1

## Data Availability

The original contributions presented in this study are included in the article and supplementary material. Further inquiries can be directed to the corresponding author. The data are not publicly available due to privacy restriction.
