# Supplementary material for: IDO2-AhR axis as central regulator of the kynurenine pathway in glioblastoma
Source: J Neurooncol. 2025 Jun 5;174(3):721–31. doi: 10.1007/s11060-025-05106-w (PMC12263720; doi:10.1007/s11060-025-05106-w)
Supplement: Supplementary file 1 — Supplementary Material 1 [file 11060_2025_5106_MOESM1_ESM.docx]

**IDO2-AhR axis as Central Regulator of the Kynurenine Pathway in Glioblastoma**

Arnaud Jacquerie^1^*, Amanda Macamo^1^, Ann Hoeben^2^, Daniëlle B.P. Eekers^3^, Alida A. Postma^4^, Maxime Vanmechelen^5,6, 7^, Frederik De Smet^5,6^, Linda Ackermans^8^, Monique Anten^9^, Maikel Verduin^2^, Kim Severens^1^, Axel zur Hausen^1^, Jan Beckervordersandforth^1^, Martinus P.G. Broen^8^

*^1^Department of Pathology, GROW School for Oncology and Reproduction, Maastricht University Medical Centre, Maastricht, The Netherlands;*

*^2^Department of Medical Oncology, GROW School for Oncology and Reproduction, Maastricht University Medical Centre, The Netherlands;*

*^3^Department of Radiation Oncology (Maastro), GROW School for Oncology and Reproduction, Maastricht University Medical Centre, Maastricht, The Netherlands;*

*^4^Department of Radiology and Nuclear Medicine, Mental Health and Neuroscience research institute, Maastricht University Medical Centre, Maastricht, The Netherlands;*

*^5^Laboratory for Precision Cancer Medicine, Translational Cell and Tissue Research Unit, Department of Imaging and Pathology, KU Leuven, Leuven, Belgium;*

*^6^LISCO—KU Leuven Institute for Single Cell Omics, KU Leuven, Leuven, Belgium;*

*^7^Department of Medical Oncology, University Hospitals Leuven, Leuven, Belgium.*

*^8^Department of Neurosurgery, School for Mental Health and Neuroscience, Maastricht University Medical Centre, Maastricht, The Netherlands;*

*^9^Department of Neurology, GROW School for Oncology and Reproduction, Maastricht University Medical Centre, Maastricht, The Netherlands;*

***Corresponding author:** Arnaud Jacquerie; Department of Pathology, GROW School for Oncology and Reproduction, Maastricht University Medical Centre, Maastricht, The Netherlands

E-mail address: [arnaud.jacquerie@mumc.nl](mailto:arnaud.jacquerie@mumc.nl)

**Supplement 1.** *List of variables explored in relation to kynurenine pathway markers*

*Patient characteristics:*

- Age at diagnosis (years)
- Gender (male/female)
- BMI (kg/m^2^)
- ECOG performance status scale (ECOG-score)
- Visceral adipose tissue (VAT) (cm^2^)
- Subcutaneous adipose tissue (SAT) (cm^2^)
- Skeletal muscle index (SMI) (cm^2^/m^2^)

*Patient history:*

- History of hypercholesterolemia (yes/no)
- History of hypertension (yes/no)
- History of type 2 diabetes (yes/no)
- History of psychiatric disorder (yes/no)
- History of cancer (yes/no)
- History of alcohol (yes/no)
- History of smoking (yes/no)

*Patient Medication:*

- Use of dexamethasone (yes/no)
- Use of anticonvulsive therapy (yes/no)
- Use of proton pump inhibitors (yes/no)
- Use of antidepressants (yes/no)
- Use of statins (yes/no)
- Use of ACE-inhibitors (yes/no)

*Systemic blood markers:*

- Leukocytes count (cells/uL)
- Neutrophils count (cells/uL)
- Lymphocytes count (cells/uL)
- Monocytes count (cells/uL)
- CRP (mg/L)
- Hemoglobin (mmol/L)
- Creatinine (umol/L)
- Platelets count (platelets/uL)
- Albumin (g/dL)

*VASARI MRI feature set:*

- Tumour location (lobe)
- Major Axis of tumour
- Side of tumour epicenter (left/right/central)
- Paraventricular tumour invasion (yes/no)
- Ependymal extension (yes/no)
- Multifocal tumour (yes/no)

**Supplement 2.** *Correlation between demographic characteristics and the expression of kynurenine pathway markers*

|  | **TDO2 expression** | |  | **IDO1 expression** | |  | **IDO2 expression** | |  | **AhR expression** | |
| --- | --- | --- | --- | --- | --- | --- | --- | --- | --- | --- | --- |
| Variable | Spearman Correlation, r (*P*) | Chi-square, Cramér's V (*P*) |  | Spearman Correlation, r (*P*) | Chi-square, Cramér's V (*P*) |  | Spearman Correlation, r (*P*) | Chi-square, Cramér's V (*P*) |  | Spearman Correlation, r (*P*) | Chi-square, Cramér's V (*P*) |
| **Age at diagnosis** | **.288 (.009)**** | NA |  | .048 (.670) | NA |  | .135 (.234) | NA |  | -.028 (.798) | NA |
| **Gender** | NA | **.279 (.012)*** |  | NA | .005 (.963) |  | NA | .046 (.681) |  | NA | .059 (.583) |
| **BMI** | .050 (.662) | NA |  | .081 (.180) | NA |  | -.064 (.581) | NA |  | -.017 (.876) | NA |
| **ECOG score** | NA | .245 (.095) |  | NA | .164 (.351) |  | NA | .0.81 (.779) |  | NA | .217 (.146) |
| **VAT** | .246 (.137) | NA |  | .309 (.056) | NA |  | .222 (.181) | NA |  | .139 (.398) | NA |
| **SAT** | -.185 (.328) | NA |  | .097 (.603) | NA |  | -.165 (.384) | NA |  | -.107 (.567) | NA |
| **SMI** | .089 (.597) | NA |  | -.050 (.762) | NA |  | .049 (.769) | NA |  | .083 (.617) | NA |

Kynurenine pathway markers were assessed in relation to demographic characteristics. The associations with categorical demographic variables were analyzed using the chi-square test, and the strength of association was quantified using Cramer's V, with corresponding *P*-values reported. For continuous demographic variables, the Spearman rank correlation coefficient was used, accompanied by the respective *P*-values. Significance levels were set at *P* < 0.05. ECOG score: ECOG performance status scale, VAT: visceral adipose tissue, SAT: subcutaneous adipose tissue, SMI: skeletal muscle index. TDO2 tryptophan 2,3-dioxygenase, IDO1 indoleamine 2,3-dioxygenase 1, IDO2 indoleamine 2,3-dioxygenase 2, AhR aryl hydrocarbon receptor, * *P* < .05, ** *P* < .01

**Supplement 3.** *Association between patients’ history and the expression of kynurenine pathway markers*

|  |  | **TDO2 expression** | |  |  | **IDO1 expression** | |  |  | **IDO2 expression** | |  |  | **AhR expression** | |
| --- | --- | --- | --- | --- | --- | --- | --- | --- | --- | --- | --- | --- | --- | --- | --- |
| Variable | Low | High | Chi-square, Cramér's V (*P*) |  | Low | High | Chi-square, Cramér's V (*P*) |  | Low | High | Chi-square, Cramér's V (*P*) |  | Low | High | Chi-square, Cramér's V (*P*) |
| **Hypercholesterolemia** |  |  |  |  |  |  |  |  |  |  |  |  |  |  |  |
| No | 35 | 34 | .099 (.376) |  | 10 | 57 | .173 (.260) |  | 21 | 47 | .006 (1.000) |  | 27 | 45 | .090 (.616) |
| Yes | 4 | 7 |  |  | 4 | 8 |  |  | 3 | 7 |  |  | 6 | 6 |  |
| **Hypertension** |  |  |  |  |  |  |  |  |  |  |  |  |  |  |  |
| No | 28 | 26 | .089 (.424) |  | 6 | 46 | .225 (.092) |  | 20 | 33 | .220 (.052) |  | 23 | 33 | .052 (.636) |
| Yes | 11 | 15 |  |  | 8 | 19 |  |  | 4 | 21 |  |  | 10 | 18 |  |
| **Cancer** |  |  |  |  |  |  |  |  |  |  |  |  |  |  |  |
| No | 35 | 39 | .102 (.625) |  | 12 | 61 | .117 (.627) |  | 23 | 49 | .088 (.750) |  | 30 | 48 | .061 (.901) |
| Yes | 4 | 2 |  |  | 2 | 4 |  |  | 1 | 5 |  |  | 3 | 3 |  |
| **DM type II** |  |  |  |  |  |  |  |  |  |  |  |  |  |  |  |
| No | 36 | 37 | .037 (.744) |  | 13 | 59 | .028 (1.000) |  | 24 | 47 | .209 (.156) |  | 32 | 44 | .178 (.211) |
| Yes | 3 | 4 |  |  | 1 | 6 |  |  | 0 | 7 |  |  | 1 | 7 |  |
| **Psychiatric disorder** |  |  |  |  |  |  |  |  |  |  |  |  |  |  |  |
| No | 33 | 37 | .085 (.673) |  | 12 | 57 | .023 (1.000) |  | 22 | 46 | .089 (.672) |  | 30 | 44 | .070 (.767) |
| **Alcohol** |  |  |  |  |  |  |  |  |  |  |  |  |  |  |  |
| No | 13 | 11 | .029 (.809) |  | 5 | 19 | .032 (1.000) |  | 9 | 15 | .057 (.636) |  | 9 | 16 | .084 (.477) |
| Yes | 23 | 22 |  |  | 8 | 36 |  |  | 14 | 30 |  |  | 21 | 26 |  |
| **Smoking** |  |  |  |  |  |  |  |  |  |  |  |  |  |  |  |
| No | 23 | 9 | .348 (.004) |  | 5 | 26 | .057 (.643) |  | 9 | 22 | .097 (.434) |  | 13 | 22 | .058 (.626) |
| Yes | 13 | 22 |  |  | 7 | 27 |  |  | 13 | 21 |  |  | 15 | 20 |  |

The table presents the distribution of patients across high and low enzyme expression groups, categorized by the presence or absence of a specific patient history. The relationship was examined using the chi-square test and Cramer's V was utilized as a measure of the strength of association, with corresponding p-values reported. Significance levels were set at *P* < 0.05. TDO2 tryptophan 2,3-dioxygenase, IDO1 indoleamine 2,3-dioxygenase 1, IDO2 indoleamine 2,3-dioxygenase 2, AhR aryl hydrocarbon receptor, * *P* < .05, ** *P* < .01

**Supplement 4.** *Association between common medication usage and the expression of kynurenine pathway markers*

|  |  | **TDO2 expression** | |  |  | **IDO1 expression** | |  |  | **IDO2 expression** | |  |  | **AhR expression** | |
| --- | --- | --- | --- | --- | --- | --- | --- | --- | --- | --- | --- | --- | --- | --- | --- |
| Variable | Low | High | Chi-square, Cramér's V (*P*) | | Low | High | Chi-square, Cramér's V (*P*) | | Low | High | Chi-square, Cramér's V (*P*) | | Low | High | Chi-square, Cramér's V (*P*) |
| **Dexamethasone** |  |  |  |  |  |  |  |  |  |  |  |  |  |  |  |
| No | 19 | 9 | **.267 (.018)*** |  | 6 | 23 | .079 (.705) |  | 5 | 22 | .190 (.098) |  | 12 | 19 | .160 (.148) |
| Yes | 20 | 30 |  |  | 7 | 41 |  |  | 18 | 31 |  |  | 24 | 28 |  |
| **Anticunvulsants** |  |  |  |  |  |  |  |  |  |  |  |  |  |  |  |
| No | 29 | 34 | .132 (.239) |  | 10 | 53 | .044 (1.000) |  | 19 | 42 | .054 (.864) |  | 30 | 35 | **.297 (.007)**** |
| Yes | 10 | 6 |  |  | 3 | 12 |  |  | 4 | 12 |  |  | 2 | 16 |  |
| **PPI** |  |  |  |  |  |  |  |  |  |  |  |  |  |  |  |
| No | 21 | 14 | .190 (.092) |  | 8 | 29 | .126 (.265) |  | 9 | 27 | .100 (.382) |  | 13 | 26 | .101 (.358) |
| Yes | 18 | 26 |  |  | 5 | 36 |  |  | 14 | 27 |  |  | 19 | 25 |  |
| **Antidepressants** |  |  |  |  |  |  |  |  |  |  |  |  |  |  |  |
| No | 37 | 34 | .164 (.280) |  | 12 | 58 | .038 (1.000) |  | 21 | 48 | .036 (1.000) |  | 29 | 46 | .007 (1.000) |
| Yes | 2 | 6 |  |  | 1 | 7 |  |  | 2 | 6 |  |  | 3 | 5 |  |
| **Statins** |  |  |  |  |  |  |  |  |  |  |  |  |  |  |  |
| No | 36 | 28 | **.284 (.011)*** |  | 10 | 53 | .044 (1.000) |  | 21 | 42 | .161 (.278) |  | 26 | 41 | .011 (923) |
| Yes | 3 | 12 |  |  | 3 | 12 |  |  | 2 | 12 |  |  | 6 | 10 |  |
| **ACE-inhibitors** |  |  |  |  |  |  |  |  |  |  |  |  |  |  |  |
| No | 32 | 33 | .006 (.958) |  | 10 | 54 | .060 (.895) |  | 21 | 44 | .124 (.457) |  | 28 | 41 | .092 (.400) |
| Yes | 7 | 7 |  |  | 3 | 11 |  |  | 2 | 10 |  |  | 4 | 10 |  |

Kynurenine pathway markers were assessed in relation to common medication use. The associations with different medications were analyzed using the chi-square test, and the strength of association was quantified using Cramer's V, with corresponding *P* -values reported. Significance levels were set at *P* < 0.05. TDO2 tryptophan 2,3-dioxygenase, IDO1 indoleamine 2,3-dioxygenase 1, IDO2 indoleamine 2,3-dioxygenase 2, AhR aryl hydrocarbon receptor, * *P* < .05, ** *P* < .01

**Supplement 5.** *Correlation between preoperative blood markers and the expression of kynurenine pathway markers*

|  | **TDO2 expression** | |  | **IDO1 expression** | |  | **IDO2 expression** | |  | **AhR Expression** | |
| --- | --- | --- | --- | --- | --- | --- | --- | --- | --- | --- | --- |
| Blood marker | Correlation coefficient (r) | Sig. (*P*) | | Correlation coefficient (r) | Sig. (*P*) |  | Correlation coefficient (r) | Sig. (*P*) |  | Correlation coefficient (r) | Sig. (*P*) |
| **Leukocytes** | -.113 | .365 |  | .319 | **.010*** |  | -.082 | .516 |  | .001 | .996 |
| **Neutrophils** | -.139 | .411 |  | .244 | .146 |  | .001 | .993 |  | .173 | .300 |
| **Lymphocytes** | -.129 | .448 |  | -.022 | .898 |  | .046 | .786 |  | -.046 | .783 |
| **Monocytes** | -.175 | .301 |  | -.033 | .846 |  | -.075 | .657 |  | -.116 | .490 |
| **CRP** | .279 | .061 |  | .301 | **.045*** |  | -.137 | .363 |  | -.349 | **.014*** |
| **Hemoglobin** | -.027 | .827 |  | .068 | .586 |  | -.018 | .883 |  | -.029 | .808 |
| **Creatinine** | -.225 | .073 |  | .301 | **.017*** |  | .092 | .475 |  | .083 | .510 |
| **Platelets** | .120 | .360 |  | -.290 | **.027*** |  | -.145 | .274 |  | -.108 | .403 |
| **Albumin** | -.351 | .394 |  | -.398 | .377 |  | .010 | .982 |  | .668 | .070 |

Kynurenine pathway markers were assessed in relation to preoperative blood markers. The Spearman rank correlation coefficient was used, accompanied by the respective p-values. Significance levels were set at *P* < 0.05. TDO2 tryptophan 2,3-dioxygenase, IDO1 indoleamine 2,3-dioxygenase 1, IDO2 indoleamine 2,3-dioxygenase 2, AhR aryl hydrocarbon receptor, * *P* < .05, ** *P* < .01

**Supplement 6.** *Association between molecular markers obtained from next-generation sequencing and the expression of kynurenine pathway markers*

|  |  | **TDO2 expression** | |  |  | **IDO1 expression** | |  |  | **IDO2 expression** | |  |  | **AhR expression** | |
| --- | --- | --- | --- | --- | --- | --- | --- | --- | --- | --- | --- | --- | --- | --- | --- |
| Variable | Low | High | Chi-square, Cramér's V (*P*) |  | Low | High | Chi-square, Cramér's V (*P*) |  | Low | High | Chi-square, Cramér's V (*P*) |  | Low | High | Chi-square, Cramér's V (*P*) |
| **MGMT methylated** |  |  |  |  |  |  |  |  |  |  |  |  |  |  |  |
| No | 26 | 33 | .115 (.303) |  | 9 | 50 | .111 (.517) |  | 16 | 41 | .051 (.651) |  | 21 | 41 | .186 (.088) |
| Yes | 12 | 9 |  |  | 5 | 15 |  |  | 7 | 14 |  |  | 12 | 10 |  |
| **EGFR amplified** |  |  |  |  |  |  |  |  |  |  |  |  |  |  |  |
| No | 18 | 20 | .013 (1.000) |  | 6 | 33 | .061 (.602) |  | 10 | 27 | .030 (1.000) |  | 15 | 27 | .050 (.659) |
| Yes | 18 | 19 |  |  | 4 | 28 |  |  | 11 | 26 |  |  | 15 | 22 |  |
| **EGFR mutated** |  |  |  |  |  |  |  |  |  |  |  |  |  |  |  |
| No | 26 | 29 | .024 (.834) |  | 8 | 46 | .119 (.497) |  | 16 | 39 | .054 (.817) |  | 21 | 37 | .061 (.591) |
| Yes | 10 | 10 |  |  | 5 | 15 |  |  | 5 | 14 |  |  | 9 | 12 |  |
| **PTEN mutated** |  |  |  |  |  |  |  |  |  |  |  |  |  |  |  |
| No | 25 | 31 | .115 (.318) |  | 9 | 45 | .039 (1.000) |  | 16 | 39 | .027 (.817) |  | 25 | 34 | 0156 (.167) |
| Yes | 11 | 8 |  |  | 4 | 16 |  |  | 5 | 14 |  |  | 5 | 15 |  |
| **P53 mutated** |  |  |  |  |  |  |  |  |  |  |  |  |  |  |  |
| No | 24 | 61 | .125 (.283) |  | 9 | 44 | .035 (1.000) |  | 15 | 39 | .014 (.902) |  | 25 | 33 | .162 (.151) |
| Yes | 11 | 8 |  |  | 4 | 16 |  |  | 5 | 14 |  |  | 5 | 15 |  |
| **TERT mutated** |  |  |  |  |  |  |  |  |  |  |  |  |  |  |  |
| No | 11 | 7 | .147 (.202) |  | 4 | 13 | .086 (.709) |  | 8 | 9 | .226 (.101) |  | 9 | 10 | .109 (.333) |
| Yes | 25 | 32 |  |  | 9 | 48 |  |  | 13 | 44 |  |  | 21 | 39 |  |

Kynurenine pathway markers were assessed in relation to molecular markers obtained from NGS. The associations with different molecular markers were analyzed using the chi-square test, and the strength of association was quantified using Cramer's V, with corresponding *P* -values reported. Significance levels were set at *P* < 0.05. TDO2 tryptophan 2,3-dioxygenase, IDO1 indoleamine 2,3-dioxygenase 1, IDO2 indoleamine 2,3-dioxygenase 2, AhR aryl hydrocarbon receptor, * *P* < .05, ** *P* < .01

**Supplement 7.** *Correlation between MR imaging characteristics of GBM using the VASARI feature set and the expression of kynurenine pathway markers*

|  |  | **TDO2 expression** | |  | **IDO1 expression** | |  | **IDO2 expression** | |  | **AhR expression** | |
| --- | --- | --- | --- | --- | --- | --- | --- | --- | --- | --- | --- | --- |
| Variable | **Analysis method** | **N** | **Test statstic (*P*)** |  | **N** | **Test statstic (*P*)** |  | **N** | **Test statstic (*P*)** |  | **N** | **Test statstic (*P*)** |
| **Tumour location (lobe)** | Kruskal-Wallis | 81 | 14.446 (.273) |  | 80 | 14.707 (.196) |  | 79 | 9.373 (.588) |  | 85 | 13.733 (.318) |
| **Major Axis of tumour** | Pearson/Spearman correlation | 81 | .089 (.555) |  | 80 | .057 (.710) |  | 79 | .003 (.986) |  | 85 | .012 (.935) |
| **Side of tumour epicenter** |  |  |  |  |  |  |  |  |  |  |  |  |
| Right | Kruskal-Wallis | 49 | **6.027 (.049)*** |  | 48 | 3.122 (.210) |  | 48 | .631 (0628) |  | 50 | 2.083 (.353) |
| Left |  | 30 |  |  | 31 |  |  | 30 |  |  | 33 |  |
| Central/Bilateral |  | 2 |  |  | 1 |  |  | 1 |  |  | 2 |  |
| **Paraventricular tumour invasion** |  |  |  |  |  |  |  |  |  |  |  |  |
| No | Pearson Chi-square | 24 | .574 (.449) |  | 23 | .402 (.757) |  | 24 | .024 (.877) |  | 26 | 1.561 (.212) |
| Yes |  | 57 |  |  | 57 |  |  | 55 |  |  | 59 |  |
| **Ependymal extension** |  |  |  |  |  |  |  |  |  |  |  |  |
| No | Pearson Chi-square | 64 | .982 (.322) |  | 63 | 2.122 (.273) |  | 63 | 1.696 (.318) |  | 67 | .012 (.914) |
| Yes |  | 17 |  |  | 17 |  |  | 16 |  |  | 18 |  |
| **Multifocal tumour** |  |  |  |  |  |  |  |  |  |  |  |  |
| No | Pearson Chi-square | 77 | .903 (.662) |  | 76 | .164 (1.000) |  | 75 | .058 (1.000) |  | 81 | .175 (1.000) |
| Yes |  | 4 |  |  | 4 |  |  | 4 |  |  | 4 |  |
| **Corpus callosum involvment** |  |  |  |  |  |  |  |  |  |  |  |  |
| No | Pearson Chi-square | 74 | .086 (1.000) |  | 74 | .003 (1.000) |  | 73 | 4.042 (.121) |  | 78 | .026 (1.000) |
| Yes |  | 7 |  |  | 6 |  |  | 6 |  |  | 7 |  |

Kynurenine pathway markers were assessed in relation to MR imaging characteristics of GBM using the VASARI features. The associations with categorical demographic variables were analyzed using the chi-square test, with corresponding P-values reported. For continuous demographic variables, the Spearman rank correlation coefficient was used, accompanied by the respective P-values. The Kruskal-Wallis test was used for categorical variables with >2 categories. Significance levels were set at *P* < 0.05. TDO2 tryptophan 2,3-dioxygenase, IDO1 indoleamine 2,3-dioxygenase 1, IDO2 indoleamine 2,3-dioxygenase 2, AhR aryl hydrocarbon receptor, * *P* < .05, ** *P* < .01

**Supplement 8.** *Correlation between TDO2, IDO1, IDO2 and AhR expression*

|  | **TDO2 expression** |  |  | **IDO1 expression** | |  | **IDO2 expression** | |  | **AhR Expression** | |
| --- | --- | --- | --- | --- | --- | --- | --- | --- | --- | --- | --- |
| Variable | Correlation coefficient (r) | Sig. (*P*) |  | Correlation coefficient (r) | Sig. (*P*) |  | Correlation coefficient (r) | Sig. (*P*) |  | Correlation coefficient (r) | Sig. (*P*) |
| **TDO2 expression** | NA | NA |  | .022 | .848 |  | .184 | .106 |  | .016 | .887 |
| **IDO1 expression** |  |  |  | NA | NA |  | .008 | .945 |  | -.052 | .649 |
| **IDO2 expression** |  |  |  |  |  |  | NA | NA |  | .532 | **<.001**** |

Expression of kynurenine pathway markers was assessed. The Spearman rank correlation coefficient was used, accompanied by the respective p-values. Significance levels were set at *P* < 0.05. TDO2 tryptophan 2,3-dioxygenase, IDO1 indoleamine 2,3-dioxygenase 1, IDO2 indoleamine 2,3-dioxygenase 2, AhR aryl hydrocarbon receptor, * *P* < .05, ** *P* < .01

**Supplement 9.** *Association between percentage of tumour cells and expression of kynurenine pathway markers*

|  | **TDO2 expression** | |  | **IDO1 expression** | |  | **IDO2 expression** | |  | **AhR Expression** | |
| --- | --- | --- | --- | --- | --- | --- | --- | --- | --- | --- | --- |
| Variable | Correlation coefficient (r) | Sig. (*P*) |  | Correlation coefficient (r) | Sig. (*P*) |  | Correlation coefficient (r) | Sig. (*P*) |  | Correlation coefficient (r) | Sig. (*P*) |
| **Tumour cells (%)** | .071 | .566 |  | .184 | .131 |  | .448 | **<.001**** |  | .089 | .469 |

The expression of kynurenine pathway markers was assessed in relation to the percentage of tumour cells. The Spearman rank correlation coefficient was used, accompanied by the respective p-values. Significance levels were set at *P* < 0.05. TDO2 tryptophan 2,3-dioxygenase, IDO1 indoleamine 2,3-dioxygenase 1, IDO2 indoleamine 2,3-dioxygenase 2, AhR aryl hydrocarbon receptor, * *P* < .05, ** *P* < .01


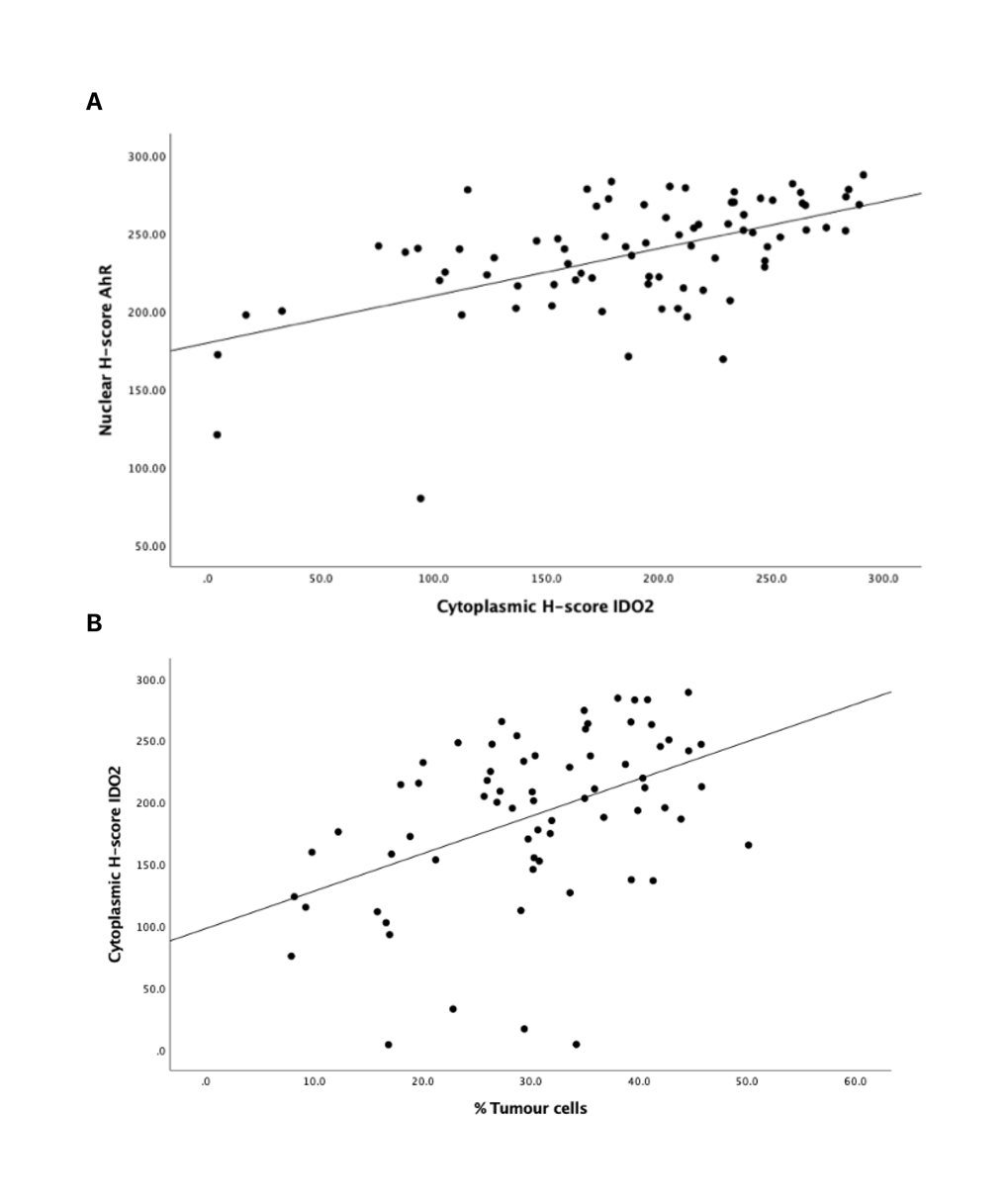

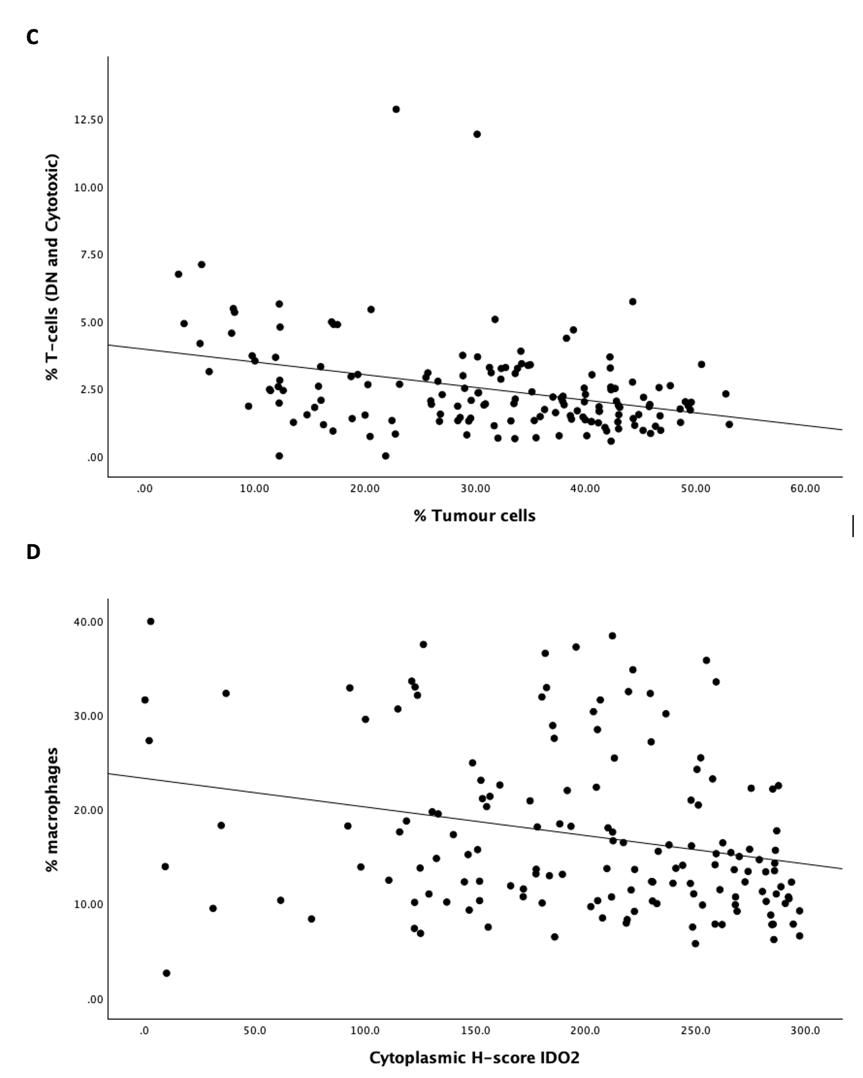


**Supplement 10.** Spatial correlations between IDO2 expression, AhR, tumor density, and immune cell infiltration in GBM

Scatterplots illustrate: (A) IDO2 expression vs AhR expression; (B) IDO2 expression vs percentage of tumor cells; (C) IDO2 expression vs percentage of macrophages; and (D) percentage of tumor cells vs percentage of T cells. These spatial correlations were derived from digital pathology analysis of multiplex immunofluorescence-stained GBM tissue. Abbreviations: IDO2, indoleamine 2,3-dioxygenase 2; AhR, aryl hydrocarbon receptor; GBM, glioblastoma.
